# Editorial: Impact of face covering on social cognition and interaction

**DOI:** 10.3389/fnins.2023.1150604

**Published:** 2023-02-21

**Authors:** Marina A. Pavlova, Claus-Christian Carbon, Yann Coello, Arseny A. Sokolov, Alice M. Proverbio

**Affiliations:** ^1^Social Neuroscience Unit, Department of Psychiatry and Psychotherapy and Tübingen Center for Mental Health, Medical School and University Hospital, Eberhard Karls University of Tübingen, Tübingen, Germany; ^2^Department of General Psychology and Methodology, University of Bamberg, Bamberg, Bavaria, Germany; ^3^Univ. Lille, CNRS, UMR 9193 - SCALab - Sciences Cognitives et Sciences Affectives, Lille, France; ^4^Service de Neuropsychologie et de Neuroréhabilitation, Département des Neurosciences Cliniques, Centre Hospitalier Universitaire Vaudois, Lausanne, Switzerland; ^5^Cognitive Electrophysiology Lab, Department of Psychology, University of Milano-Bicocca, Milan, Italy

**Keywords:** face coverage, social cognition, emotion, social neuroscience, mental disorders, psychotherapy, social interaction, body language

## Facemasks within and beyond the pandemic

Facemasks have become a familiar item due to the COVID-19 pandemic, but there is a multitude of further face coverings, with which we are confronted day-to-day and which impact our social cognition and interaction, for instance, scarves, headscarves, or bandanas. Such items might be used as protection, symbols of religious faith, or due to a mere fashion aspect, but all of them cover parts of a face, evidently reducing the overall amount of information we can gather. As coverings can be used as symbolic items, they can be psychologically charged, leading not only to a possible loss of information and shifts of attention but also to a potential adding of associations, perceptual biases, and prejudices.

**Figure 1:**
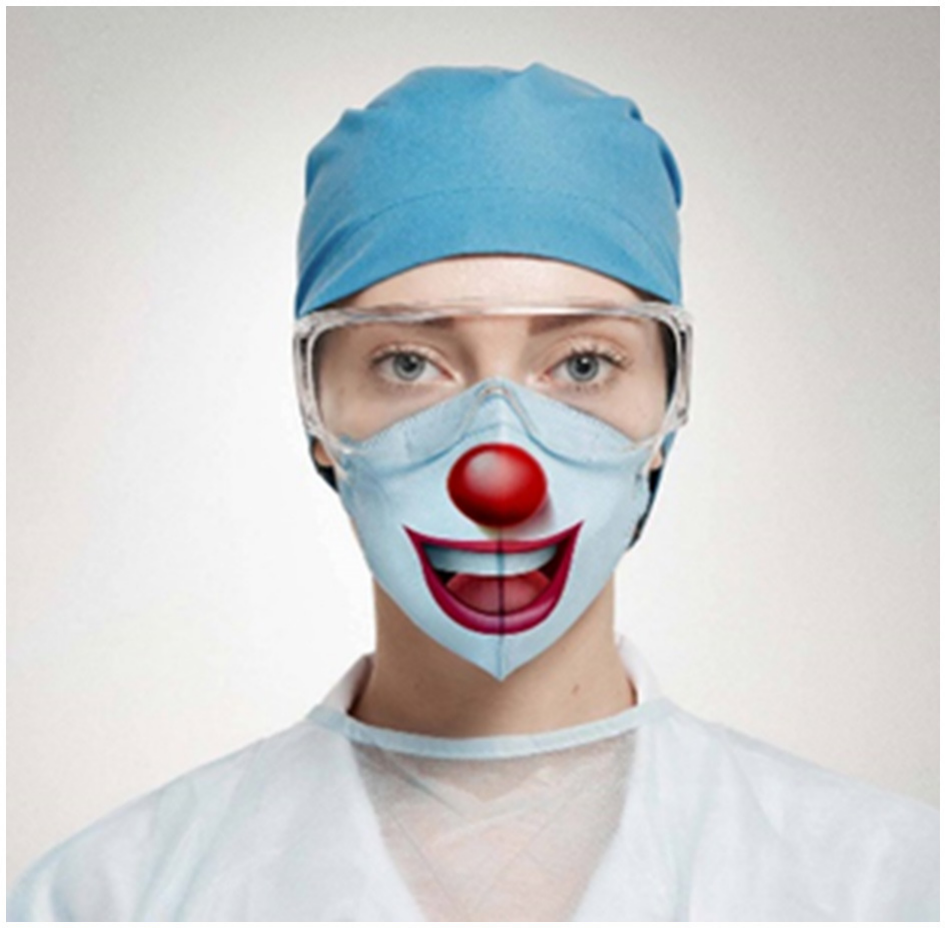
As shown by this Research Topic, facemasks affect emotion recognition and social interaction. Yet people easily differentiate between true and social (fake, dishonest) smiles even when covered by facemasks. Better no smile at all than a fake smile (Pavlova and Sokolov, 2022a). Some attempts to diminish the negative effects, in particular, in the healthcare system, require special rigorous experimenting. The image (courtesy of Jyo John Mulloor, a digital artist) is reproduced with his written permission.

In this Research Topic (https://www.frontiersin.org/research-topics/29292/impact-of-face-covering-on-social-cognition-and-interaction), 18 articles with 77 authors circling around this theme were edited by the international and interdisciplinary team of researchers and health professionals. The contributions came primarily from different parts of Western Europe and Canada. The Research Topic is urgently needed as, currently, we do not understand a full range of consequences elicited by a face covering that affect our mental processes, including perception, emotions, social cognition, and communication. While it seems that face coverings consistently impair the confident reading of facial expressions, recognition of identities, and the ability to understand speech from visual inputs, it is less understood how face coverings affect other aspects of social interaction (Figure 1). This will give answers to the questions of how they affect trustworthiness, perceiver's attitudes toward covered persons, or, for instance, how perceived gaze direction changes when looking at a covered face. The articles compiled for this Research Topic will provide a platform for joint interdisciplinary discussions and approaching the theme from different research perspectives and methodologies.

## Facial masking affects emotion recognition and social attribution

More traditional line of research is related to examination of the impact of facemasks on the recognition of emotions and social attribution (such as trustworthiness or attractiveness) by using static face photographs with superimposed on them masks. Overall, these studies nicely dovetail with the initial research outcome (Carbon, 2020; Cartaud et al., 2020; Biermann et al., 2021; Calbi et al., 2021; Gori et al., 2021; Grundmann et al., 2021; Kamatani et al., 2021; Marini et al., 2021, for comprehensive review, see Pavlova and Sokolov, 2022a). Yet only a handful of studies implements more ecologically valid dynamic faces (Leitner et al.; Aguillon-Hernandez et al.). Proverbio and Cerri report that although face masking reduces emotion recognition by about 30%, not all emotions are negatively affected. Face covering is most detrimental to sadness and disgust, while recognition of anger remains relatively intact. The authors speculate that facial masking polarizes non-verbal communication toward the happiness/anger dimension, while minimizing the impact of subtle emotions on empathic responses in the observer. Noteworthy, rather similar effects are demonstrated using dynamic faces: masks significantly impede the perception of disgust and sadness in videos of face expressions, whereas recognition of fear, neutral expressions, and social (fake, dishonest) smiling remains largely intact (Leitner et al.). Irrespective of facemasks, in dynamic faces, true Duchenne smiles are perceived as more honest than social smiles. In general, this is in line with earlier work with static faces. Even covered by masks true smiles are rated as happy and pleasant, in other words, *the glow of real smiles still shows* (Sheldon et al., 2021; see also Pavlova and Sokolov, 2022a). Unexpectedly, social fake smiles appear more honest in masked than in unmasked faces. Verroca et al. show that facemasks reduce the perceived intensity of facial expressions (except for extreme fear), and the ability to recognize subtle expressions, such as moderate fear and disgust. These detrimental effects, particularly for disgust (very often misinterpreted as anger), but also for happiness and sadness, do not seem to be reduced by habituation or learning. Indeed, Carbon et al. have demonstrated no improvement in the ability to recognize facial expressions after 1 year of surgical mask usage among the population. Face masking affects not only reading of face language, but also speech recognition. By using videos of dynamic faces, Aguillon-Hernandez et al. show that surgical masks impair the recognition of happiness and sadness (but not neutral expressions), as well as of spoken bilabial syllables. Mask covering appears not only to impair effective communication, but also to alter emotional transfer between people and social attribution processes. Leder et al. presented photographs of individuals in daily situations with and without masks, asking participants to evaluate their attractiveness, liking, and character. Persons wearing masks were perceived as more attractive and valuable by people with strong positive attitudes toward protective devices. In agreement with earlier reports (see review by Pavlova and Sokolov, 2022a), facemasks were found to impair the ability to evaluate people's trustworthiness and personal traits (Cannito et al.), on which economical transactions (e.g., risky choices) are commonly based. In summary, face covering alters many social processes.

## Impact of face covering on social interaction

Studying interpersonal spatial adjustments, Geers and Coello find that social and peripersonal spaces are interconnected with a preference for shorter distances in females compared to males. Wearing a face mask induces shorter social distances primarily in persons with high aversion to risks and germs, which the authors interpret as an influence of the behavioral immune system on social interactions. Thomas et al. report that facemasks enhance significance of extraneous information such as head orientation and gaze direction, in particular, for emotions poorly recognized with a mask. Furthermore, facemasks make the eyes more noticeable, which leads to several perceptual biases. Lobmaier and Knoch show that mutual gaze is not recognized more accurately in masked faces, whereas Liu et al. report that facemasks induce a wider range of gaze angles associated with mutual gaze perception, increasing the feeling of “*being looked at*.” This highlights social significance of a gaze potentially causing inappropriate social behavior. Villani et al. demonstrate that under conditions associated with an approaching behavior, wearing a mask forces people to jointly orient visual attention in the direction of a seen gaze. By using videos of moving faces, Rabadan et al. show that a facemask alters visual exploration of faces, with less time spent in its lower part, but preserves pupil reactivity to facial expressions. They conclude that although facemasks impair emotion recognition, implicit physiological responses to facial expressions remain unchanged. Overall, these studies reveal that facemask wearing may alter some aspects of perception of non-verbal social cues, in particular, those usually used to adjust interpersonal behavior in various social contexts.

## Impact of facemasks is diminished by other social signals

For achieving efficient daily-life social interaction during the COVID-19 pandemic, we are forced to combine social signals from different sources such as the eyes (with a face hidden behind a mask) and bodies. Clarifying the issue of how facemasks affect face reading in real life, where we deal with dynamic faces and have access to additional social signals such as body language, warrants rigorous experimental work (Pavlova and Sokolov, 2022a). In real life, we usually cope with plentiful and often redundant social information that helps to prevent paying high costs for maladaptive social interaction, and, therefore, conceivably diminishes effects of masks. In accord with this, Ross and George report that the negative impact of masks on recognition of facial emotions (anger, happiness, fear, and sadness) becomes negligible for all emotions (except for happiness) when a whole body with a congruent static posture is visible. Nevertheless, with masks, confidence levels are lower for all emotions despite an additional body information. Moreover, Pavlova et al. show that in males, reading language of the eyes (when the overall amount of available information is rather comparable with that in faces covered by masks) is knotted with reading of dynamic point-light faces, while in females, inferring emotions from dynamic point-light bodies and faces are firmly linked. Amazingly, in males only, accuracy of the eyes, face, and body reading was found to be negatively tied with autistic traits. This outcome further underscores gender-specific modes in reading covered faces as well as reading language of the eyes (Pavlova and Sokolov, 2022b). On the same wavelength, McCrackin and Ristic demonstrate that the negative impact of masks on judgment of emotional valence and intensity in static faces (depicting happiness and sadness) is lessened by the availability of a larger emotional context, for instance, prior presentation of written statements such as “*Her pet cat was found yesterday afternoon*.”

## Face masks in mental disorders and during psychotherapy

Reading covered faces may be particularly challenging for individuals with neuropsychiatric conditions characterized by aberrant non-verbal social cognition already in the pre-pandemic period (Pavlova and Sokolov, 2022a). In one of the pioneering studies conducted by Escelsior et al., among patients with major depressive disorder (MDD), schizophrenia (SZ), bipolar disorder (BD), and typically developing individuals, patients with MDD and SZ were found to experience most difficulties in identifying subtle expressions of happiness. Erschens et al. present the outcome of a survey in patients (*N* = 66) and healthcare professionals (*N* = 33): (i) whereas patients report the impact of masks on individual psychotherapy and relationships with psychotherapists to be low, facemasks have greater subjectively estimated effects on the interaction group therapy; and (ii) negative effects of facemasks on therapeutic treatment are reported more frequently by professionals.

## Limitations and further directions

In a nutshell, the work presented in this Research Topic nicely dovetails with and enriches the outcome of initial studies. Alongside a more traditional and widespread line of research on face covering effects on emotion recognition in static faces and social interaction, there are also ground-breaking studies on dynamic faces as well as the influence of context and other social signals (such as bodies) and ties between them. However, there is still a lack of developmental (including healthy aging), cross-cultural and brain imaging work, in particular, in psychiatric and neurological populations. The most research remains online with its well-known advantages (in particular, during the pandemic), but also rather serious limitations. As reported by the first comprehensive analysis on the topic (Pavlova and Sokolov, 2022a), online studies may create a sampling bias (e.g., study samples are usually heavily predominated by young women) precluding a proper generalization of findings. In addition, standardization of visual input is limited in some studies: faces with different emotions substantially differ not only in facial information *per se*, but also in head tilts and face angles. Other boundaries already mentioned earlier currently remain as well, namely: (i) displayed expressions (by performers asked to demonstrate) instead of natural *truly felt* emotions; and (ii) basic emotions instead of complex mental states. The most promising asset to future research and intervention appears to be an assessment of facemasks impact on social interaction and cognition in *daily-life* situations.

## Author contributions

MAP conceptualized the structure of the manuscript, elaborated suggestions made by C-CC, YC, AAS, and AMP, placed together all portions, and drafted the manuscript. All authors substantially contributed to the article by writing manuscript sections and editing the paper, and approved the submitted version.
